# All-systolic first-pass myocardial rest perfusion at a long saturation time using simultaneous multi-slice imaging and compressed sensing acceleration

**DOI:** 10.1002/mrm.28712

**Published:** 2021-03-10

**Authors:** Giulio Ferrazzi, Sarah McElroy, Radhouene Neji, Karl P. Kunze, Muhummad Sohaib Nazir, Peter Speier, Daniel Stäb, Christoph Forman, Reza Razavi, Amedeo Chiribiri, Sébastien Roujol

**Affiliations:** 1 School of Biomedical Engineering and Imaging Sciences, Faculty of Life Sciences and Medicine, King’s College London, London, United Kingdom; 2 IRCCS San Camillo Hospital, Venice, Italy; 3 MR Research Collaborations, Siemens Healthcare Limited, Frimley, United Kingdom; 4 Cardiovascular MR predevelopment, Siemens Healthcare GmbH, Erlangen, Germany; 5 MR Research Collaborations, Siemens Healthcare Limited, Melbourne, Australia

**Keywords:** all-systolic myocardial rest perfusion, compressed sensing, dark rim artefact, perfusion contrast, simultaneous multi-slice

## Abstract

**Purpose:**

To enable all-systolic first-pass rest myocardial perfusion with long saturation times. To investigate the change in perfusion contrast and dark rim artefacts through simulations and surrogate measurements.

**Methods:**

Simulations were employed to investigate optimal saturation time for myocardium-perfusion defect contrast and blood-to-myocardium signal ratios. Two saturation recovery blocks with long/short saturation times (LTS/STS) were employed to image 3 slices at end-systole and diastole. Simultaneous multi-slice balanced steady state free precession imaging and compressed sensing acceleration were combined. The sequence was compared to a 3 slice-by-slice clinical protocol in 10 patients. Quantitative assessment of myocardium-peak pre contrast and blood-to-myocardium signal ratios, as well as qualitative assessment of perceived SNR, image quality, blurring, and dark rim artefacts, were performed.

**Results:**

Simulations showed that with a bolus of 0.075 mmol/kg, a LTS of 240470 ms led to a relative increase in myocardium-perfusion defect contrast of 34% ± 9%-28% ± 27% than a STS = 120 ms, while reducing blood-to-myocardium signal ratio by 18% ± 10%-32% ± 14% at peak myocardium. With a bolus of 0.05 mmol/kg, LTS was 320-570 ms with an increase in myocardium-perfusion defect contrast of 63% ± 13%-62% ± 29%. Across patients, LTS led to an average increase in myocardium-peak pre contrast of 59% (*P* < .001) at peak myocardium and a lower blood-to-myocardium signal ratio of 47% (*P* < .001) and 15% (*P* < .001) at peak blood/myocardium. LTS had improved motion robustness (*P* = .002), image quality (*P* < .001), and decreased dark rim artefacts (*P* = .008) than the clinical protocol.

**Conclusion:**

All-systolic rest perfusion can be achieved by combining simultaneous multi-slice and compressed sensing acceleration, enabling 3-slice cardiac coverage with reduced motion and dark rim artefacts. Numerical simulations indicate that myocardium-perfusion defect contrast increases at LTS.

## 1 Introduction

First-pass contrast enhanced myocardial perfusion^1^ is 1 of the techniques used in cardiovascular magnetic resonance (CMR) examinations, allowing for the detection of areas of inducible myocardial ischemia and the diagnosis of coronary artery disease (CAD).^2,3^ During a perfusion exam, a pharmacological stress agent is administered to the patient to induce a difference in the perfusion rate of healthy and ischemic myocardium. Dynamic CMR acquisitions during first-pass injection of gadolinium-based contrast agents are used to visualize the differences in contrast arrival time and tissue enhancement, which correspond to the heterogeneities in perfusion.

Conventional CMR perfusion sequences are based on dynamic imaging using multi-slice 2D single-shot electrocardiogram-t riggered saturation recovery sequences. A typical protocol encompasses the acquisition of (at least) 3 slices planned in short axis orientation within each R-R interval. A short shot duration (from 100 ms to 150 ms) is commonly employed to “freeze” cardiac motion, thus limiting image blurring. Images can be acquired using gradient echo (GRE), hybrid EPI, or a balanced steady-state free precession (bSSFP) readout. Although there is no consensus on the optimal readout type, bSSFP has higher SNR/contrast-to-noise ratio (CNR) properties, which is particularly attractive at lower fields such as 1.5 Tesla. International guidelines recommend the use of an in-plane resolution < 3 × 3 mm^2^.^4^ However, the use of higher spatial resolution is desirable to reduce dark rim artefacts, which are a major confounding factor for the detection of subendocardial perfusion defects,^5^ and to enable reliable estimation of transmural perfusion gradients—a predictor of hemodynamically significant CAD.^6^

In conventional CMR perfusion sequences, all slices are acquired sequentially and thus at different cardiac phases. This can augment in-plane cardiac motion artefacts and partial volume effects.^7^ Moreover, this problem may be more pronounced toward the apex in patients with arrhythmias^8^ or thin myocardial wall.^9^

The delay between the saturation pulse and the k-space center along the phase-encoding (PE) direction, often referred to as the saturation delay time (TS), is a key parameter gov-erning contrast between healthy and ischemic myocardium. A longer/shorter TS can modulate the presence of dark rim artefacts, which are caused by sharp signal transitions at the boundaries between blood and myocardium^5,10^ in combination with Gibbs ringing. The clinical need for acquiring multiple slices, however, constrains TS to be short (typically 80-120 ms). The use of a longer TS could be beneficial to increase myocardium-perfusion defect (M-PD) contrast, which in turn is expected to improve the detection of perfusion defects and to reduce dark rim artefacts by means of a lower blood-to-myocardium (BM) signal ratio (see Supporting Information Figure S1).

Such constraints (uncontrolled cardiac phase and suboptimal TS) could be relaxed by acquiring all slices in 1 shot at a prespecified cardiac phase. 3D acquisitions enable increased spatial coverage within a shot^11^ but are associated with limited spatial resolution and longer shot time (about 200 ms). Alternatively, simultaneous multi-slice (SMS) imaging using multiband RF pulses enables the acquisition of multiple slices simultaneously in nearly the same time as a conventional single slice acquisition. This results in increased spatial coverage without loss in spatial and temporal resolution.^12^ SMS techniques have been successfully applied to cardiac perfusion^13–17^ because the SNR is higher when compared to conventional parallel imaging acceleration.^18,19^ SMS makes use of controlled aliasing in parallel imaging results in higher acceleration (CAIPIRINHA) encoding to facilitate slice separation.^20^ SMS CAIPIRINHA has been optimized for bSSFP using a dedicated phase cycling scheme^19^ and gradient-controlled local Larmor adjustment (GC-LOLA) to reduce sensitivity to off-resonance.^21^ This approach has been demonstrated for dynamic first-pass perfusion imaging and offers doubled slice coverage with preserved in-plane resolution.^16,17^ SMS can be further combined with compressed sensing (CS)^22^ to enable higher acceleration factors and improved spatial resolution.^18^

In this study, we present a first-pass myocardial perfusion imaging sequence which, making use of SMS bSSFP, GC-LOLA, and CS, facilitates the acquisition of 3 slices at the end-systolic phase with a long saturation time. The concept provides 3 systolic slices at the same cardiac phase. To investigate possible changes in perfusion contrast and BM signal ratio as a result of the longer TS, a second diastolic acquisition block is acquired after end-systole but with a short TS. Simulation results are provided to investigate optimal settings for M-PD contrast and BM signal ratios. The sequence was used to acquire data in 10 patients without suspected CAD. Myocardium-peak pre (M-PP) contrast as a surrogate for M-PD contrast was calculated and compared across conditions.

## 2 Methods

### 2.1 Proposed pulse sequence

**Figure 1:**
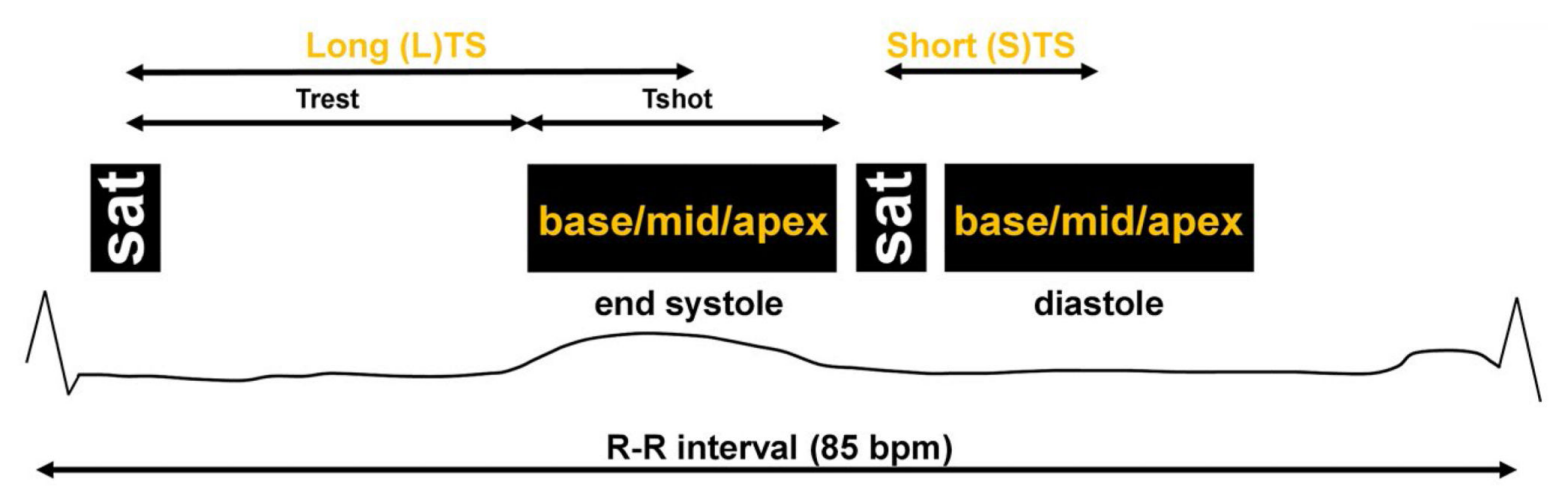
Sequence diagram for the proposed scheme. Acquisition of base, mid-, and apical slices is performed simultaneously at end-systole using a long TS and at diastole using a short TS. The time variable Trest and Tshot are reported to facilitate the reading of Numerical Simulation (subsection 2.5.1 of this article). Sequence elements are displayed for a target heart rate of 85 bpm. bpm, beats per minute; Trest, time between saturation pulse and the beginning of the shot; TS, saturation delay time

The pulse diagram of the proposed sequence is shown in Figure 1. It consists of 2 main acquisition blocks within each R-R interval. In each block, a saturation pulse is applied before the simultaneous acquisition of 3 slices (base, mid-, and apex) using SMS bSSFP^19^ with GC-LOLA^21^ and CS undersampling.^22^ To enable 2 measurements within the heartbeat, the same set of 3 slices is acquired twice, once within each block. The delay between the saturation pulse and the center of the shot differs in the 2 blocks. For the first block, this is set to be a long TS (LTS) to ensure that imaging happens during a quiescent period of the cardiac cycle (end-systole). The second block instead uses a short TS (STS) enabling the acquisition of an additional set of slices in diastole. The acquisition scheme is repeated across consecutive heartbeats for the duration of the perfusion scan.

### 2.2 SMS3 bSSFP with GC-LOLA

In the following, it is omitted that the MR signal from all slices is subject to transmit–receive phase cycling increments of π to center the frequency response of the bSSFP signal onto the Larmor frequency.

**Figure 2:**
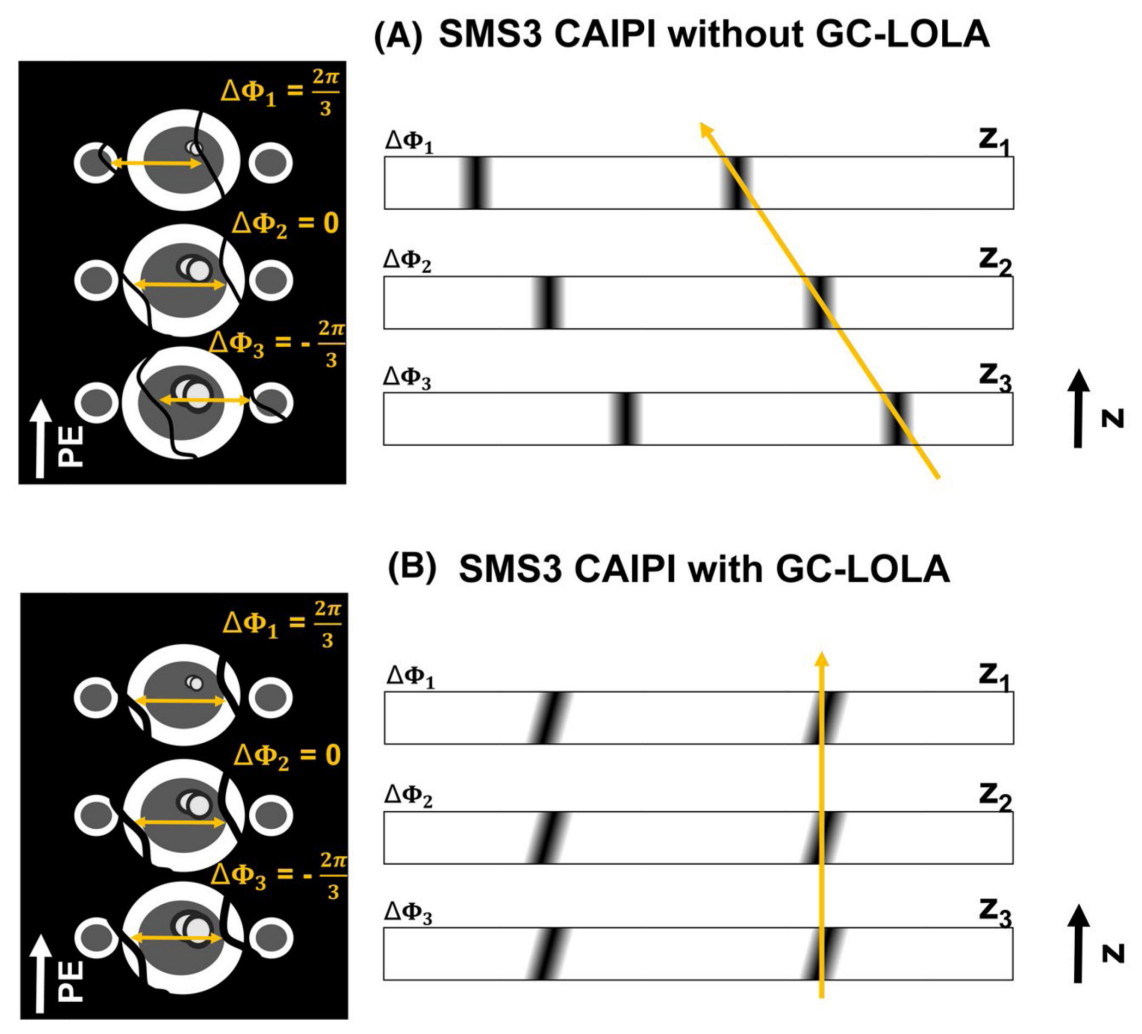
Schematic representation of the SMS framework. (A) Left: SMS3 with CAIPIRINHA encoding but without GC-LOLA. The application of CAIPIRINHA RF phase increments $\Delta \phi_{1}=\frac{2\pi }{3}$ and $\Delta \phi_{3}=-\frac{2\pi }{3}$ determines spatial shifts along the PE direction of apical and basal slices of FOV/3 and –FOV/3. When combining CAIPIRINHA encoding with bSSFP imaging, the bSSFP frequency response of apex and base is also shifted by ± a third of the total bandwidth with respect to the mid-slice (orange arrows). Right: schematic through-plane representation of the bSSFP frequency response for bands 1, 2, and 3 at positions z_1_ z_2_, and z_3_ with CAIPIRINHA RF phase increments Δ*ϕ*_1_, Δ*ϕ*_2_, and Δ*ϕ*_3_. The shifts in the frequency response across slices are represented with an orange arrow. (B) GC-LOLA correction with SMS3. After the application of Δ*M_LOLA_* and Δ*ϕ_LOLA_* from Equations (1) and (2), all bands are aligned irrespective from their position along the *z* axis (orange arrow). bSSFP, balanced steady-state free precession; CAIPIRINHA, controlled aliasing in parallel imaging results in higher acceleration; GC-LOLA, gradient-controlled local Larmor adjustment; PE, phase encoding; SMS, simultaneous multi-slice

The SMS bSSFP GC-LOLA framework, originally validated with a multiband factor of 2 (SMS2),^21^ has been extended to enable a multiband factor of 3 (SMS3). Three triple-band RF pulses with CAIPIRINHA phase increments $\Delta \phi_{1}=\frac{2\pi }{3}$, Δ*ϕ*_2_ = 0, and $\Delta \phi_{3}=-\frac{2\pi }{3}$ were generated as the complex summation of a native sinc-shaped single band pulse (bandwidth time product = 1.6). This achieves shifts in image space of FOV/3, 0, and –FOV/3 for slices 1, 2, and 3 (schematic representation in Figure 2A, left), which has been shown to improve the separation of the slices^20^ with respect to SMS with no shifts applied.^12^ However, because each band is subject to independent RF phase cycling schemes, the frequency response of the bSSFP signal is subject to slice-specific undesirable shifts.^19^ For the case SMS3 in combination with the proposed CAIPIRINHA encoding strategy, these are equal to 1/3, 0, and –1/3 of the native bSSFP pass-band interval (schematic representation in Figure 2A, right).

This effect was removed using the GC-LOLA frame-work^21^ by 1) applying an additional slice unbalancing gradient (ΔM_LOLA_) within each TR to align the frequency response of the bands, and 2) employing an additional phase cycling term (Δ*ϕ*_LOLA_) to center the frequency response of the bands onto the water peak. For the case SMS3, and taking as reference slices 1 and 2 at positions z_1_ and z_2_ from Figure 2, these quantities are calculated as:

(1) $$\Delta {\text{M}}_{\text{LOLA}}=\frac{\Delta \phi_{1}-\Delta \phi_{2}}{\gamma \left({\text{z}}_{1}-{\text{z}}_{2}\right)}$$

and

(2) $$\Delta \theta_{\text{LOLA}}=\frac{\Delta \phi_{2}{\text{z}}_{1}-\Delta \phi_{1}{\text{z}}_{2}}{{\text{z}}_{1}-{\text{z}}_{2}},$$

where *γ* is the gyromagnetic ratio. For a detailed description please refer to Ref. 21.

### 2.3 CS undersampling scheme

An undersampling scheme has been developed to satisfy the following constraints: 1) promote spatiotemporal incoherence of the sampling pattern, 2) maintain the bSSFP steady state signal as well as slice shifts across the FOV, and 3) limit large jumps in k-space to reduce eddy current-induced artefacts.

This has been achieved by extending the SMS2 bSSFP CS undersampling scheme developed in Ref. 17 as follows. The first step is to divide the k-space sampling lattice into 3 regions: 1 central region and 2 peripheral ones. The central region is fully sampled, whereas the 2 peripheral regions are undersampled. Undersampling of each peripheral region is performed independently. First, all phase-encoding line indices are binned into 3 groups. Each group corresponds to lines excited by 1 of the 3 multiband pulses (ie, group 1 contains k-space locations 1,4,7…, group 2 locations 2,5,8 …, and so on). Then, a percentage of the k-space lines, inversely proportional to the in-plane acceleration factor, is chosen at random to form 3 subgroups of equal size. To limit large jumps in k-space, each subgroup is sorted in ascending order. The final k-space trajectory is generated by sequentially selecting the first line from each subgroup until all k-space locations are included. The overall k-space trajectory starts with the upper peripheral region, followed by the fully sampled central region and the lower peripheral region. This algorithm is used to generate a different undersampling pattern for each dynamic. For a detailed description of the achieved point spread function, please refer to Ref. 17.

### 2.4 Reconstruction

The CS reconstruction uses a nonlinear iterative formulation with spatiotemporal regularization in the wavelet domain.^23^ It is formulated as follows:

(3) $$\begin{array}{c} {\left\{x_{t}\right\}}_{t=1,\ldots ,T}=\text{arg}{\text{min}}_{\left\{x_{t}\right\}}\sum_{t=1}^{T}\left({\left\|A_{t}x_{t}-y_{t}\right\|}_{2}^{2}+\lambda_{\omega }{\left\|W_{\omega }x_{t}\right\|}_{1}\right)\\ +\lambda_{\tau }{\left\|W_{\tau }{\left\{x_{1}^{T},\ldots ,x_{T}^{T}\right\}}^{T}\right\|}_{1} \end{array}$$

where x_t_ is the image to be reconstructed at time frame *t* and A_t_ is the encoding operator, thus comprising coil sensitivities in image space, Fourier transforms, and pseudorandom sampling mask in k-space. y_t_ is the measured data at time frame *t*. W_ω_ and W_τ_ are spatial and temporal wavelet transformations, with λ_ω_ and λ_τ_ representing spatial and temporal regularization parameters. λ_ω_ and λ_τ_ were empirically optimized to 0.00005 and 0.0005, respectively. However, note that to favor an equivalent level of regularization that accounts for the different signal levels achieved at LTS and STS, the regularization parameters λ_ω_ and λ_τ_ are scaled at the beginning of the reconstruction according to maximum image intensity of a (surrogate) time-averaged dataset. This is done independently for the LTS data and STS data.

Coil sensitivity information was retrieved in the form of 10 separate free-breathing spoiled GRE scans run before the SMS3 CS scan. Spatial resolution was matched to the SMS3 CS acquisition. As in Refs. 24 and 25, these calibration data were averaged in time to reduce respiratory artefacts.

The separation of the slices was performed using the “lean” CAIPIRINHA framework.^26^ For the case SMS3, acquisition and reconstruction of the slices are based on a threefold extended FOV in PE direction. A final reconstruction step splits the resulting joint image.

### 2.5 Experimental validation

#### 2.5.1 Numerical simulation

Numerical simulations were employed to investigate the relationship between TS, BM signal ratio, and M-PD contrast. Simulations used the acquisition parameters of the SMS3 CS sequence (see next section). After application of the saturation pulse (M_z_ = 0), the longitudinal magnetization recovers toward equilibrium $\left({\text{M}}_{\text{z}}=1-{\text{e}}^{-\frac{{\text{T}}_{\text{rest}}}{{\text{T}}_{1}}}\right)$. This equation was employed for increasing values of the variable T_rest_, which represents the time between saturation pulse and the beginning of the shot (see Figure 1).

Because the k-space acquisition strategy employed in this study is hybrid linear (section 2.3 of this article), to reliably simulate tissue contrast it is important to consider the effect of the multiband RF pulses acting on the magnetization during the first half of the shot (see variable T_shot_ in Figure 1). To achieve this, the recovered magnetization before the application of the first RF pulse is fed into the Bloch equations to simulate the evolution of the transverse magnetization through the (first half) of the bSSFP module (note that 6 startup pulses with linearly increasing flip angles were employed to stabilize the bSSFP signal at the beginning of the shot, and these were simulated as well). The simulated signal was measured as the absolute value of the transverse magnetization at k-space center (*k_PE_* = 0). Taken as a whole, the simulated range for the saturation time $TS={\text{T}}_{\text{rest}}+\frac{{\text{T}}_{\text{shot}}}{2}$ was 100 ms-600 ms.

To calculate T_1_/T_2_ values of the myocardium, blood, and defect signals, we adopted the following procedure: All simulations assumed the injection of Gadovist (Bayer, Berlin, Germany), with relaxivity values r_1_ = 4.7 L/(mmol*s) and r_2_ = 6.8 L/(mmol*s).^27^ The relationship between gadolinium contrast concentration *Gd* and the myocardial/blood relaxation rates $R_{1}=\frac{1}{{\text{T}}_{1}}$ and $R_{2}=\frac{1}{{\text{T}}_{2}}$ is *R*_1{*Gd*}_ = *R*_1{0}_ + *r*_1_ * *Gd* and *R*_2{*Gd*}_ = *R*_2_{0} + *r*_2_ * *Gd* where *R*_1{*Gd*}_ and *R*_2{*Gd*}_ are the myocardial/blood relaxation rates at contrast concentration *Gd,* and *R*_1{0}_ and *R*_2{0}_ are the native myocardial relaxation rates (*Gd* = 0). *R*_1{0}_ and *R*_2{0}_ are calculated considering native myocardial T_1_ and T_2_ values of 1200 ms and 50 ms,^28^ as well as native blood T_1_and T_2_ values of 1500 ms and 250 ms.^29^ Reference contrast concentration values for *Gd* were set according to stress perfusion conditions.^30^ For the myocardium, peak myocardium (peak myo) signal conditions were calculated twice assuming a *Gd* of 0.75 mmol/L and 0.49 mmol/L, which for healthy myocardium correspond to a T_1_ of 229/319 ms and T_2_ of 40/43 ms, respectively. Note that the value of 0.49 mmol/L was derived from^30^ considering a bolus contrast injection of 0.05 mmol/kg. The value of 0.75 mmol/L was extrapolated from Ref. 30 with a 0.075 mmol/kg dose. To simulate peak blood signal conditions, the modelled left ventricular blood pool contrast concentration *Gd* had a range of 1-6.5 mmol/L,^31^ corresponding to a T_1_ range of 186-32 ms and a T_2_ range of 93-21 ms.

M-PD contrast was calculated as the signal difference between defect and healthy myocardium signals at peak myo signal enhancement. In particular, for the healthy myocardium this corresponds to fixed T_1_/T_2_ values of 229/40 ms and 319/43 ms for 0.75/0.49 mmol/L gadolinium contrast concentrations (see above). For the defect signal, contrast concentration ranged from 0 mmol/L (ie, no perfusion of the contrast agent into the defect, leading to maximum perfusion contrast) to 0.75/0.49 mmol/L (ie, healthy myocardium, leading to no perfusion contrast). The calculated values were divided by those obtained with a short saturation time (120 ms) to obtain a relative M-PD measure (M-PD rel.).

The BM signal ratio was taken as an indicator for dark rim artefacts (ie, such artefacts will not be generated when the ratio between blood and myocardial signals is 1) (Supporting Information Figure S1). This was calculated at peak blood (native myocardial T_1_/T_2_ = 1200/50 ms, assuming no perfusion in the myocardium yet) and peak myo (myocardial T_1_/T_2_ = 229/40 ms).

#### 2.5.2 In vivo acquisition

The sequence was evaluated in 10 patients (6 female, mean age 44 ± 18 years) referred for clinical nonstress contrast-enhanced CMR at 1.5T (MAGNETOM Aera, Siemens Healthcare, Erlangen, Germany) using an 18-element body coil and a 32-channel spine coil. This study was approved by the National Research Ethics Service (15/NS/0030), and written informed consent was obtained from all patients. Each subject underwent 2 rest perfusion scans using the proposed sequence and a conventional perfusion sequence (see below), separated by at least 10 min to allow for contrast washout. The order was randomized across subjects. A dose of 0.075 mmol/kg of Gadovist (Bayer) was administered during each perfusion experiment. Patients were asked to perform an exhale breath hold during first-pass perfusion. For the proposed sequence, the first block had a LTS which was adjusted from cine acquisitions to capture end-systole (minimum/maximum across patients 260/370 ms). For the second block, STS was 120 ms. Imaging parameters for SMS3 CS were: FOV = 360 × 360 mm^2^, slice thickness = 10 mm, slice gap = 20 mm, resolution = 2 × 2 mm^2^, TR = 2.9 ms, TE = 1.5 ms, flip angle α = 40°, readout bandwidth = 1015 Hz/Px, start-up pulses = 6, shot duration = 155 ms. A CS undersampling factor R_CS_ with an effective in-plane acceleration of 3.33 was employed.^26^ SMS3 bSSFP with GC-LOLA was used to image base, mid-, and apex (R_SMS_ = 3). Taken as a whole, total acceleration was R_tot_ = R_SMS_*R_CS_ ≃ 10.

The conventional perfusion scans were acquired using a single-band saturation recovery bSSFP sequence accelerated in-plane by a factor of 3 employing temporal GRAPPA (TGRAPPA).^32^ Saturation delay time (TS = STS = 120 ms), spatial resolution, slice thickness, slice gap, FOV, and receive bandwidth were matched to the proposed sequence, leading to slight adjustments of the following parameters: TR = 2.7 ms, TE = 1.4 ms, shot duration = 161 ms.

Finally, to provide initial evidence of the potential benefits offered by LTS versus STS acquisitions, myocardium-to-scar contrast as surrogate for perfusion contrast was considered in 2 patients that presented chronic myocardial scar. The first patient was scanned using the SMS3 CS protocol defined above and the second using a variant (SMS TGRAPPA^33^). These results are discussed in detail in Supporting Information Figures S2 and S3.

#### 2.5.3 In vivo evaluation

The 2 sets of SMS images were compared in terms of M-PP contrast and BM signal ratio. Because none of the first 10 patients was referred for the assessment of ischemia, perfusion defects were not expected; therefore, baseline myocardial signal (ie, prior to arrival of contrast agent) was used as a surrogate for M-PD. The baseline signal was measured at peak blood signal enhancement of the right ventricle. M-PP contrast was calculated as the difference between the average myocardial signal at peak myo and the average baseline myocardial signal. A relative score was then calculated by referencing the M-PP at LTS to STS = 120 ms. This relative measure is called *M-PP rel.*

BM was calculated as the signal ratio of the left ventricular blood pool and myocardial signals at peak blood and at peak myo signal enhancement.

Subjective assessment of perceived SNR, image quality, image blurring, and dark rim artefacts was performed using the conventional sequence and the proposed sequence. All anonymized images were exported in the DICOM format and visualized using Osirix (Pixmeo SARL, Geneva, Switzerland). Two experienced cardiologists (M.S.N. and A.C., with more than 5 and 15 years of experience in CMR, respectively) assessed each dataset in consensus. Note that reviewers were blinded to clinical findings and to the aim of the analysis. Perceived SNR was scored using a 4-point scoring scale as 0: poor SNR resulting in nondiagnostic image quality, 1: major noise level but not limiting diagnosis, 2: minor noise level but not limiting diagnosis, 3: high SNR. Note that for the perceived SNR, only 1 measure per dataset was retrieved, whereas a slice-by-slice evaluation was performed for all quantities that follow. Image quality was evaluated as: 0: severe artefacts/non diagnostic, 1: major artefacts but of diagnostic quality, 2: minor artefacts and of diagnostic quality, 3: excellent/no artefacts. Dark rim artefacts and image blurring were each scored as: 0: absent, 1: present. These binary scores were summed across the 3 slices. Thus, a score of 0 to 3 signified that none to all 3 slices were affected.

#### 2.5.4 Statistical analysis

All results are reported as mean ± SD, unless stated otherwise. Differences between the LTS and STS data in terms of M-PP contrast and BM signal ratio were assessed using Wilcoxon signed rank tests, with statistical significance defined at *P* < .05. Differences between the 3 types of distributions were assessed using the Kruskal-Wallis test, and a *P* value less than .05 indicated statistical significance. If the Kruskal-Wallis test found statistical significance, Wilcoxon signed rank tests were performed for each pair of image type using Bonferroni correction.

## 3 Results

### 3.1 Numerical simulations

**Figure 3:**
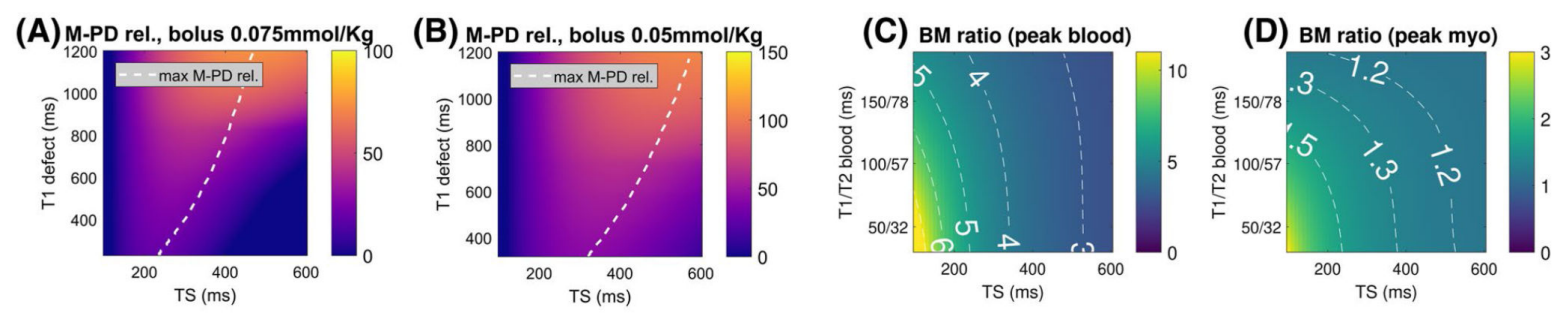
Impact of TS on M-PD rel. and BM signal ratio evaluated in numerical simulations. (A) and (B) M-PD rel. plotted as a function of TS and defect T_1_ times at peak myocardium with bolus injections of 0.075 mmol/kg and 0.05 mmol/kg, respectively. The dashed line highlights the TS/T_1_ defect combination with highest M-PD rel. (C) and (D) BM signal ratio calculated as a function of TS and blood T_1_/T_2_ times at simulated peak blood and peak myocardium, respectively. BM, blood-to-myocardium signal ratio; M-PD, myocardium-perfusion defect contrast; M-PD rel., relative M-PD contrast

The influence of TS on M-PD rel. and BM signal ratio is represented in Figure 3A,B. For the achieved myocardial defect T_1_/T_2_ values (range 229 ms/40 ms to 1200 ms/50 ms in Figure 3A; range 319 ms/43 ms to 1200 ms/50 ms in Figure 3B), M-PD rel. is maximized using TS within a range of 240-470 ms and 320-570 ms considering a bolus contrast injection of 0.075 mmol/kg and 0.05 mmol/kg (dashed white line). If we take as reference a bolus of 0.075 mmol/kg, long saturation times of 240 and 470 ms would lead to an average increase for M-PD rel. of 34% ± 9% and 28% ± 27% across the simulated T_1_/T_2_ range, respectively. Finally, with a bolus of 0.05 mmol/kg, saturation times of 320 and 570 ms lead to an average increase of M-PD rel. of 63% ± 13% and 62% ± 29%.

The T_1_/T_2_ range of the blood modelled for the BM simulations was 32 ms/21 ms to 186 ms/93 ms. BM signal ratio was always found to decrease for increasing TS values, and this was more pronounced for peak blood than peak myo (Figure 3C,D). Simulations with a TS of 200 ms and 470 ms (as previously optimized for M-PD rel. with a bolus of 0.075 mmol/kg) led to an average decrease of BM signal ratio of 32% ± 9% and 57% ± 9%, respectively, at peak blood, and 18% ± 10% and 32% ± 14% at peak myo when compared to a conventional TS of 120 ms.

### 3.2 In vivo evaluation in patients

**Figure 4:**
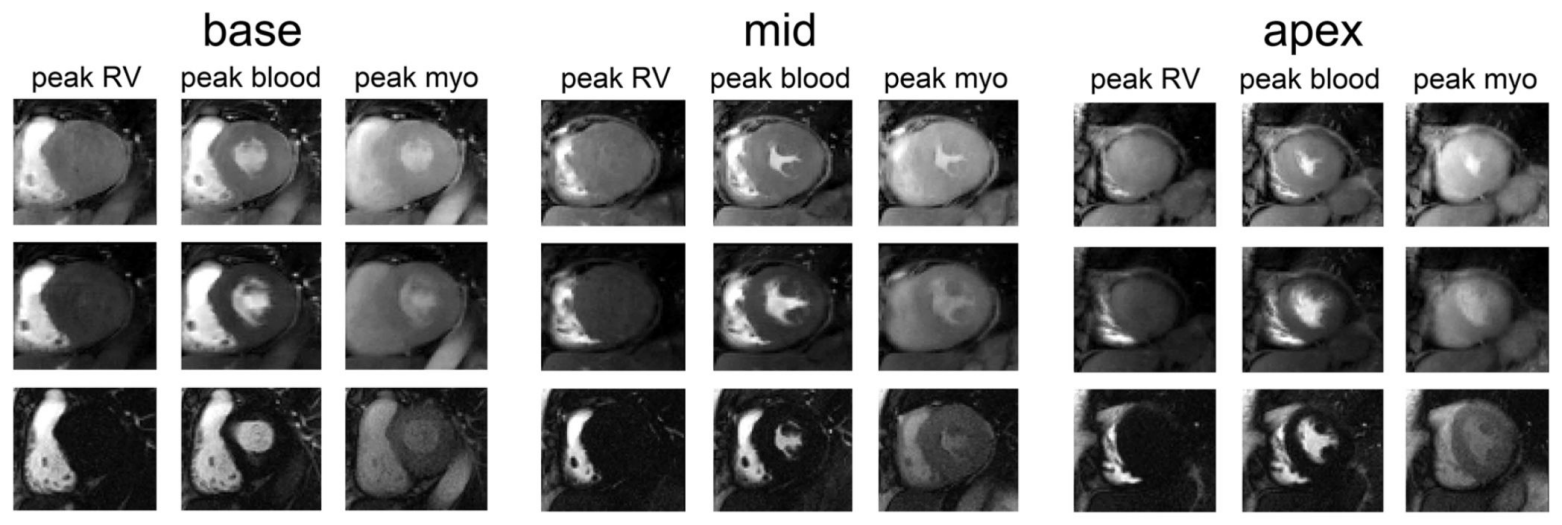
Data from 1 exemplary patient. Left to right: base, mid-, and apical slices. Top to bottom: LTS and STS data from the SMS3 CS acquisition and the TGRAPPA sequence. In each panel, data at peak blood in the right ventricle (hence no perfusion in the left ventricle), peak blood and peak myo in the left ventricle are shown. Note that in this case the TGRAPPA scan was run before SMS3 CS. CS, compressed sensing; LTS, long saturation time; SMS, simultaneous multi-slice; STS, short saturation time; TGRAPPA, temporal GRAPPA

Figure 4 shows representative images acquired in 1 patient using the proposed and TGRAPPA sequences. An animation of the data in Figure 4 is provided as Supporting Information Video S1. Comparable image quality can be observed between both techniques, whereas SMS3 CS leads to higher perceived SNR at every temporal frame. Each slice acquired with TGRAPPA corresponds to a different cardiac phase, whereas SMS3 CS enabled the acquisition of all slices at end-systole. A second set of diastolic images is also retrieved within the same heartbeat interval. Acquisition of LTS data using SMS3 CS enables imaging of all 3 slices at end-systole, thereby reducing partial volume effects. This improvement was particularly evident for the most apical slice.

**Figure 5:**
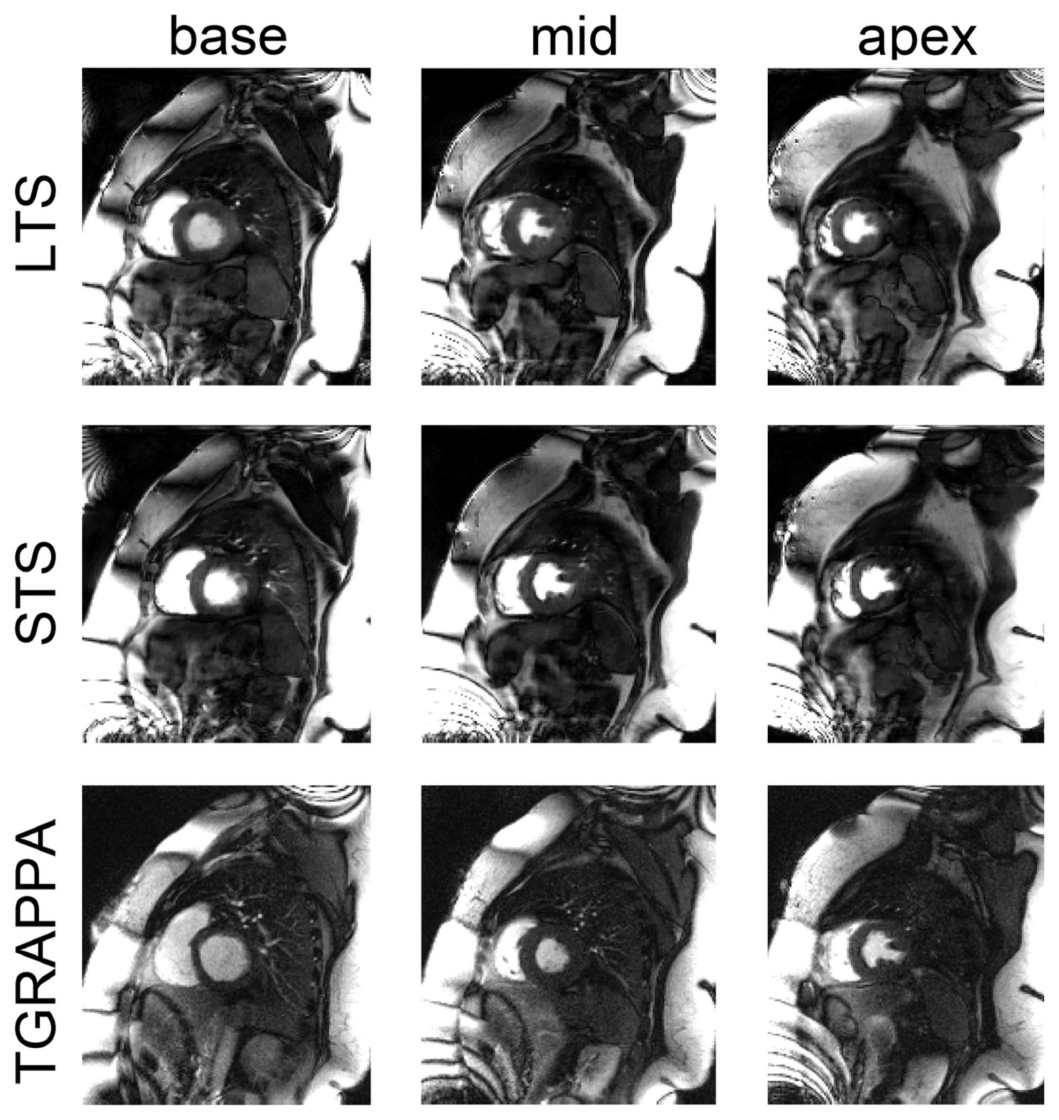
Data from 1 large patient. Reconstruction of the entire FOV of base (left), mid-, (middle), and apical (right) slices. Top and middle rows report LTS and STS data from the SMS3 CS acquisition, whereas the bottom row shows the TGRAPPA sequence. All data are shown at peak blood signal enhancement in the left ventricle. LTS, long saturation time; SMS, simultaneous multi-slice; STS, short saturation time; TGRAPPA, temporal GRAPPA

Similar findings were observed when imaging larger patients, as shown in Figure 5, illustrating the robustness of the technique in the presence of a large region with high fat signal.

**Figure 6:**
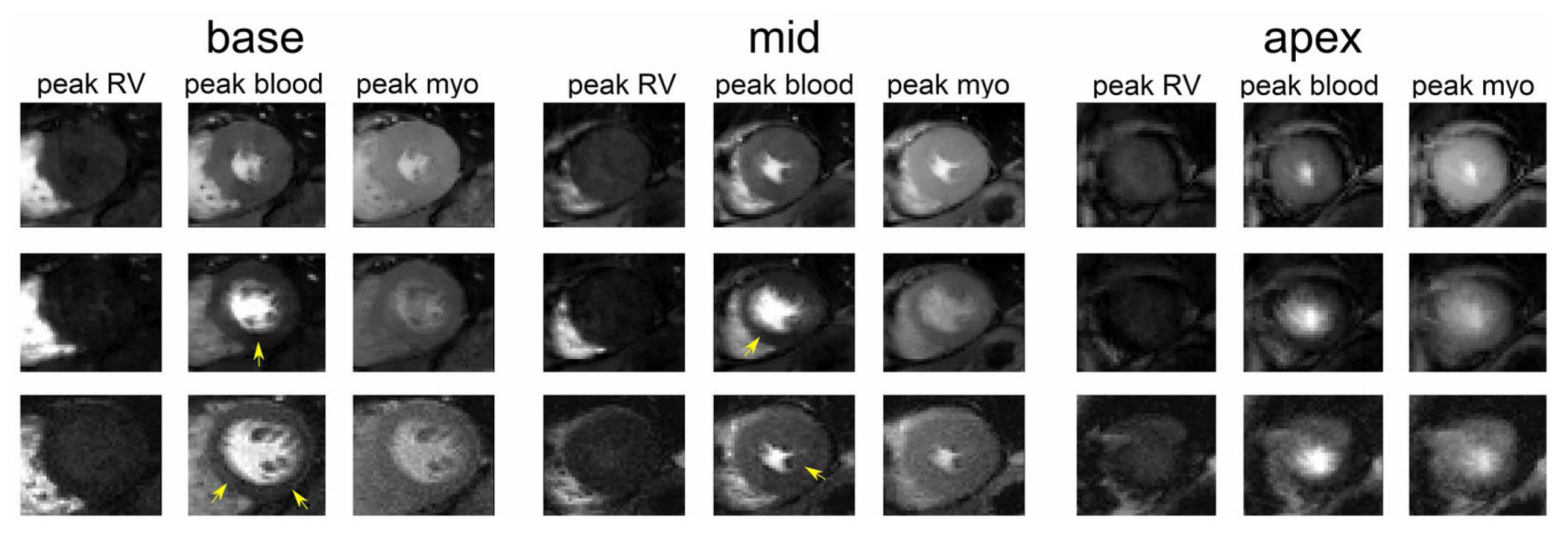
Same as in Figure 4 but from another patient. Arrows highlight the location of dark rim artefacts. Note that in this case the SMS3 CS scan was run before TGRAPPA. SMS, simultaneous multi-slice; TGRAPPA, temporal GRAPPA

Figure 6 shows another example with dark rim artefacts observed in the TGRAPPA and STS images (in both basal and mid-ventricular slices, yellow arrows). These were not present in the LTS images. An animation of the data in Figure 6 is provided as Supporting Information Video S2.

**Figure 7:**
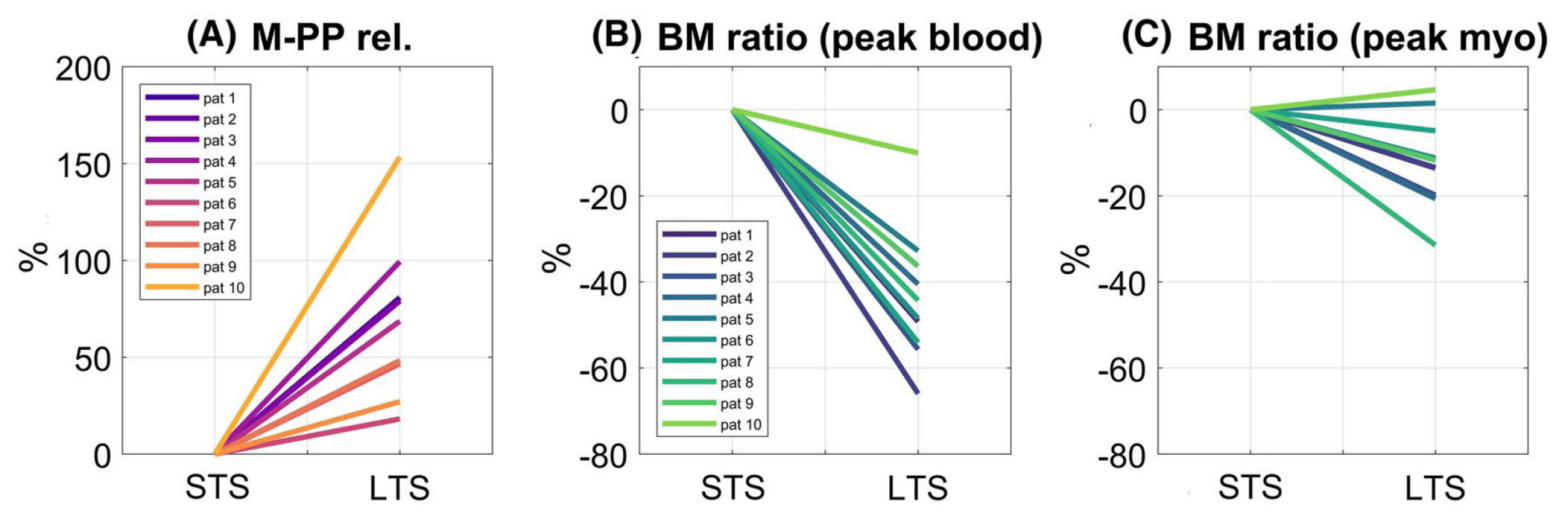
Percentage signal change in (A) M-PP rel., (B) BM signal ratio at peak blood, and (C) BM signal ratio at peak myo of LTS with respect to STS data in all patients. Values were averaged across base, mid-, and apex for each patient. BM, blood-to-myocardium signal ratio; LTS, long saturation time; M-PP, myocardium-peak pre contrast; M-PP rel., relative M-PP contrast; STS, short saturation time

Figure 7 reports M-PP rel. and BM signal ratio of LTS relative to STS images in all patients. LTS images led to a 59% increase in M-PP rel. over STS images (*P* < .001). LTS images also led to a 47% relative reduction of BM signal ratio compared to STS images at peak blood (*P* < .001) and 15% reduction at peak myo (*P* < .001).

**Figure 8:**
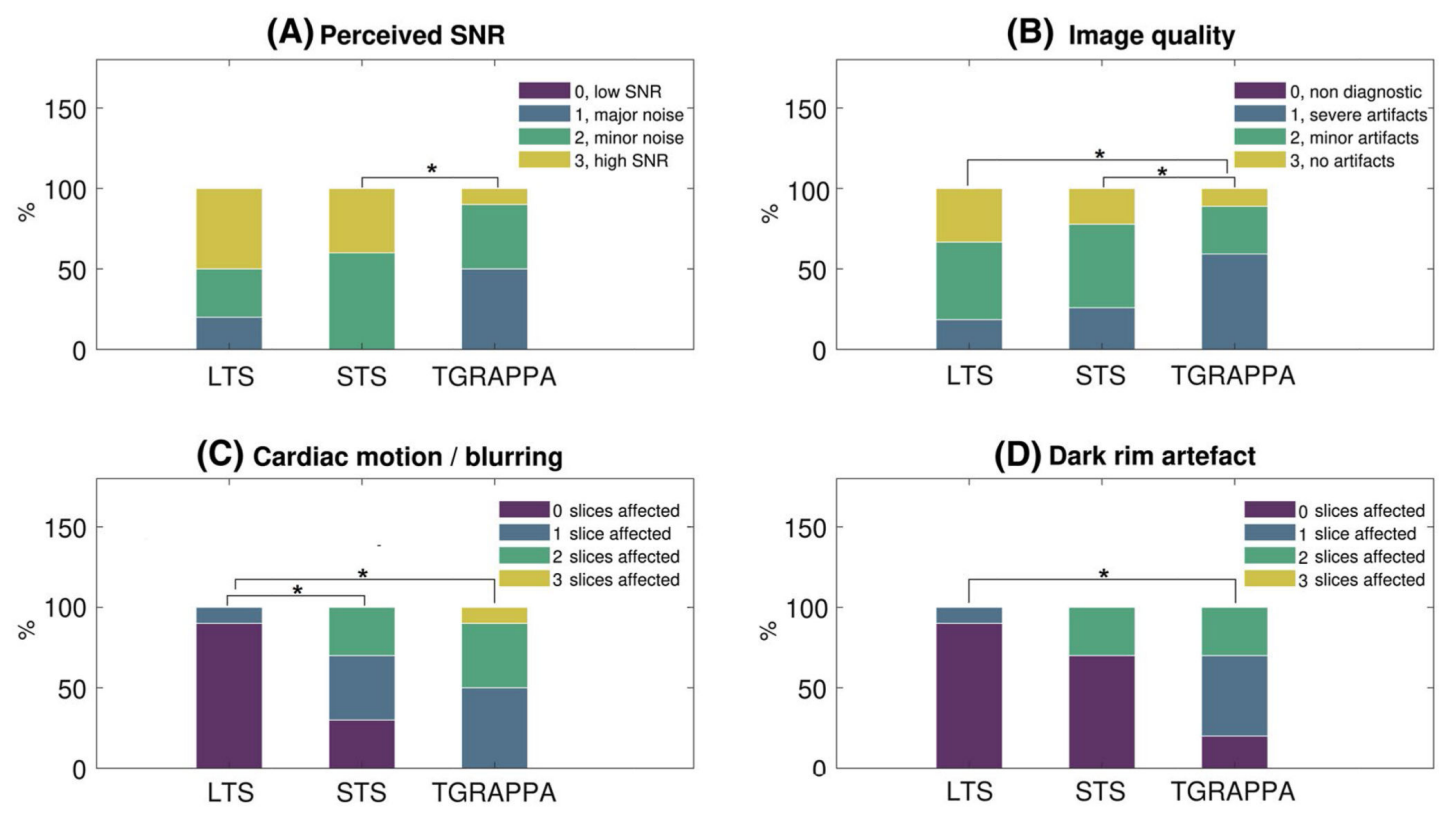
Subjective assessment of LTS, STS, and TGRAPPA datasets in 10 patients. Percentage of scores in each sequence for (A) perceived SNR. Scores were 0: low SNR resulting in nondiagnostic image quality, 1: major noise level but not limiting diagnosis, 2: minor noise level but not limiting diagnosis, 3: high SNR. (B) Image quality. Scores were 0: severe artefacts/non diagnostic, 1: major artefacts but of diagnostic quality, 2: minor artefacts and of diagnostic quality; 3: excellent/no artefacts. (C) Motion artefacts/blurring and (D) dark rim artefacts. In both (C) and (D), scores were 0: no slices, 1: one out of three, 2: two out of three, and 3: all slices affected. *denotes statistically significant differences between pairs. LTS, long saturation time; STS, short saturation time; TGRAPPA, temporal GRAPPA

Normalized frequency of subjective scores obtained using LTS, STS, and TGRAPPA datasets are shown in Figure 8. Perceived SNR of STS against TGRAPPA data was significantly higher (3.4 ± 0.51 vs. 2.6 ± 0.7, *P* = .016). Perceived SNR of LTS (3.3 ± 0.82) tends to be higher than TGRAPPA (2.6 ± 0.7), although this difference did not reach statistical significance (*P* = .12). There were no differences between LTS and STS in terms of perceived SNR (*P* = 1). Image quality was higher with LTS (3.21 ± 0.73) and STS (2.97 ± 0.68) than TGRAPPA scans (2.48 ± 0.69, *P* < .001 and *P* = .005, respectively), but there were no statistically significant differences between LTS and STS (*P* = .12). Cardiac motion/image blurring of LTS data (0.1 ± 0.32) was lower than in STS (1 ± 0.82, *P* = .016) and TGRAPPA (1.6 ± 0.7, *P* = .002), but there were no differences between STS and TGRAPPA images (*P* = .19). Finally, dark rim artefacts were detected less frequently in LTS versus TGRAPPA scans (0.10 ± 0.32 vs. 1.1 ± 0.74, *P* = .008), whereas there were no differences between STS (0.6 ± 0.97) and either LTS (*P* = .25) or TGRAPPA (*P* = .19).

There were no statistically significant differences between basal, mid-, and apical slices in terms of image quality for each of LTS (*P* ≅ 1), STS (*P* ≥ .25), and TGRAPPA (*P* ≥ .11) in all possible comparisons. The percentage of subjects classified as being affected by cardiac motion/image blurring and dark rim artefacts as a function of the excited slice is reported in Table 1. Image blurring using systolic LTS versus diastolic STS was detected 3% versus 37% of the times, respectively. Note that in the STS images, these artefacts were more present in apical images (60% of subjects) than midventricular (10%) and basal (30%) slices. TGRAPPA data had also a high rate of cardiac motion/image blurring in the apical slice (90%) and lower rates at base and mid-slice (30% and 10%). LTS had a low rate of cardiac motion/image blurring in all slices (≤ 10%).

In Supporting Information Figures S2 and S3, we report preliminary results obtained on scar positive patients. In both cases, when comparing LTS vs. STS data, we observed a relative increase of myocardium to scar contrast of 130% and 73%, respectively.

## 4 Discussion

In this study, a myocardial perfusion sequence that provides 3 systolic slices with a long saturation time using SMS acceleration, bSSFP imaging, and CS undersampling has been proposed. A second diastolic acquisition block with a short saturation time is acquired within the same heartbeat and used as control to calculate M-PP contrast and BM signal ratio. The proposed sequence led to an increase in perceived SNR and improved image quality when compared to a TGRAPPA perfusion sequence. LTS images also resulted in reduced dark rim artefacts and reduced blurring artefacts when compared against TGRAPPA. Finally, LTS images had higher M-PP contrast and reduced BM signal ratio than the STS images.

A variety of methods have been proposed to improve the efficiency of myocardial perfusion sequences.^34,35^ In this study, it was possible to accommodate 2 acquisition blocks of 3 slices per heartbeat by combining SMS3 CAIPIRINHA encoding with bSSFP imaging. This could also be achieved using alternative acceleration techniques based on SMS GRE imaging^13–15,18,36^ or 3D imaging.^11,37^ However, because SMS GRE may lead to reduced SNR/CNR at 1.5T and 3D imaging to limited spatial resolution, SMS bSSFP was selected for this study. SMS bSSFP was combined with GC-LOLA to realign the frequency response of all slices on resonance. Although this technique results in widening of dark bands,^21^ this did not impact the image quality because no banding artefacts were identified near the heart region. An alternative to SMS bSSFP GC-LOLA is the use of SMS bSSFP in combination to blipped CAIPIRINHA encoding.^38^ Although the latter technique does not suffer from widening of the dark bands as GC-LOLA,^21^ it is potentially more sensitive to chemical shift/fat leakage artefacts across slices,^39^ eddy current effects,^40^ and flow-induced artefacts.^41^ A head-to-head comparison of the 2 techniques in the context of cardiac imaging remains to be investigated.

Previous CMR perfusion studies have suggested potential benefits of performing systolic over diastolic acquisitions, including the following: a compact view of the myocardium, reduced dark rim artefacts, reduced partial volume of the apical regions, and robustness to arrhythmias due to the lower sensitivity to R-R internal variations.^8,9^ In this study, image blurring in systolic LTS was detected less often than in the STS images, especially in the apex where partial volume effects are more pronounced. In particular, the percentage of pixels in the myocardium affected by partial volume is higher in diastolic images than in systolic images (which offers a more compact view of the myocardium and thicker wall). However, the STS data were acquired immediately after the systolic STS, and it was not specifically triggered to mid-rest diastole. Thus, it is possible that cardiac motion might have played a role in the assessment of image blurring in the STS data. However, the fact that blurring was detected more frequently in the apical section, which moves less than the mid/basal sections, suggests that the partial volume may be the dominant factor.

The proposed framework could also allow the acquisition of a single mid-rest diastolic LTS dataset per heartbeat. Although mid-rest imaging could be sensitive to increased partial volume effects due to the thinner wall structure, it is generally associated with a longer quiescent resting phase than the systolic rest period, which could be beneficial to reduce the influence of cardiac motion. Further studies are needed to compare the clinical value of both approaches.

In this work, the same set of 3 slices in scanner coordinates (see below) were acquired in the LTS and STS blocks. However, the sequence could be modified to achieve full heart coverage with 3 saturation recovery imaging blocks, each with a short TS, leading to the acquisition of 9 slices. The acquisition of more slices could also contribute to improved diagnostic accuracy of CAD. Therefore, further studies are needed to compare the performance of the proposed approach against a scheme with extended slice coverage and reduced TS.

This study was performed at 1.5T. Its application at higher field strengths would require further investigation due to the increased sensitivity to off-resonance artefacts and stronger limitations on the maximum achievable flip angle imposed by specific absorption rate limits and maximum power constraints. The use of SMS GRE imaging could prove useful at higher fields.

In this work, M-PP contrast values have been reported instead of M-PP CNRs. CNR depends on the level of noise in the final images. In this study, reliable noise measurements could not be undertaken because estimation of noise is generally not straightforward when nonlinear reconstruction algorithms are used.17 It was instead assumed that STS and LTS data exhibit identical noise properties. This assumption seems reasonable because both STS and LTS datasets were acquired as part of the same acquisition using identical read-out parameters and coil settings. Therefore, the observed relative increase in M-PP contrast in LTS against STS images is expected to correspond to a comparable increase in M-PP CNR. Note that equivalent considerations should apply to M-PD contrast versus M-PD CNR.

Patients were instructed to breath-hold during the first pass of the contrast agent to avoid motion-induced artefacts caused by the temporal regularization of the CS reconstruction. The proposed approach uses a CS reconstruction, which includes temporal regularization. The latter increases the sensitivity of the sequence to motion, which has potential to result in blurring/ghosting artefacts, especially under failed breath-hold or free breathing conditions, as previously described.^16^ In this study, all data were acquired under breath-hold conditions, and the temporal regularization parameter was kept low to reduce the impact of potential motion while maintaining good image quality. Although some level of motion could be observed at the onset/end of the acquisition (see example in Supporting Information Video S2), it did not significantly affect image quality during first pass. In the future, a free-breathing acquisition strategy would be desirable, but this would require the use of a more advanced motion compensation techniques, possibly integrating prospective motion correction, motion-robust CS reconstructions, or both.

Within the proposed acquisition framework, the arterial input function quantification from LTS images is likely to be challenging due to significant recovery of the blood magnetization and may require the use of a dual sequence or dual bolus approach.

Although the main focus of this study was all-systolic myocardial perfusion, the dual contrast acquisition scheme enabled imaging of 3 slices at end-systole and diastole. By considering all possible LTS times (range 322 ± 32 ms), the acquisition window varied within 602 ± 32 ms. Thus, the highest acceptable heart rate is 100 ± 5 bpm, which should make the sequence compatible with stress conditions. The joint use of systolic and diastolic information could increase confidence in reporting perfusion defects because cross comparisons of the 2 datasets are possible. This could be beneficial in the context of wider adoption of CMR perfusion, where diagnostic accuracy is highly dependent on the reader experience.^45^ A larger clinical study is now warranted to demonstrate this potential benefit.

This study has several limitations: We chose a relatively large phase FOV of 360 mm common to all scans to ensure that it would encompass the full extent of the body even in large patients (see Figure 5). However, in clinical applications, it would be advantageous to optimize the phase FOV each time to reduce the shot duration and/or to increase the spatial resolution. Although the same slice location in scanner coordinates was acquired twice in the LTS and STS acquisition blocks, the heart moves between scans, and thus the anatomical content within a slice may have changed. Prospective motion correction techniques^42,43^ may prove useful in this context.

The trigger delay in this study was selected from cine data. Thus, the precision in systole selection is mainly determined by the cine temporal resolution (about 40 ms), possibly with some level of bias coming from the operator performing this task.

The longer saturation time of the LTS images should have translated into higher perceived SNR with respect to the STS images. However, this comparison did not reach statistical significance. We attribute the observed effect to the nonlinearity of our reconstruction framework, which employs spatiotemporal regularization that has a strong denoising effect in both LTS/STS images.

The LTS images resulted in a lower BM signal ratio and perceived dark rim than the STS images. However, LTS and STS data were acquired at a different cardiac phase, which thus may have influenced the perception of dark rim. Nevertheless, the simulations presented in Supporting Information Figure S1 suggest that a lower BM signal ratio is associated with a reduction of dark rim.

A 10 min delay was employed between the 2 rest perfusion scans. This is not sufficient to allow for the complete wash out of the contrast agent from the myocardium. To minimize the effect that this had on our measurements, the acquisition order of the 2 sequences was randomized across patients.

The baseline myocardial signal was used as a surrogate for the defect signal. Clearly such an assumption is not an accurate representation of ischemia because the baseline frames represent total blockage with no collaterals. In this study, we did not recruit CAD patients because the performance of the sequence during stress conditions remains to be investigated. This is relevant as the adoption of a stress perfusion protocol is required for the diagnosis of CAD.^46^ Thus, it remains to be assessed whether the increase in M-PP contrast during rest conditions corresponds to higher M-PD contrast at stress. In this context, our simulation results are encouraging because they predict higher perfusion contrast at longer saturation time.

## 5 Conclusion

SMS3 CS enables all-systolic myocardial rest perfusion imaging with improved perceived SNR and image quality with respect to conventional TGRAPPA acceleration. LTS images acquired at end-systole resulted in reduced dark rim artefacts, reduced blurring artefacts and better image quality when compared to TGRAPPA scans, and had a lower BM signal ratio than the STS images. Numerical simulations confirmed that M-PD contrast increases at longer saturation delay times. Future studies will assess the capability of the sequence to improve the detection of perfusion defects in CAD patients undergoing stress perfusion.

## Supplementary Material

F1

Supplementary Videos

## Figures and Tables

**Table 1:** Percentage of slices affected by residual motion–blurring artefacts/dark rim artefacts from the subject assessment as a function of the slice index and imaging modality

% of Motion-Affected Datasets/Dark Rim Artefacts	LTS	STS	TGRAPPA
Base	0%/0%	30%/20%	60%/70%
Mid	0%/0%	10%/30%	10%/10%
Apex	10%/10%	60%/10%	90%/30%

## Data Availability

The data that support the findings of this study are available from the corresponding author (S.R.) upon reasonable request.
