# Supplementary material for: All-systolic first-pass myocardial rest perfusion at a long saturation time using simultaneous multi-slice imaging and compressed sensing acceleration
Source: Magn Reson Med. Author manuscript; Available in PMC 2022 Feb 1. (PMC7611406; doi:10.1002/mrm.28712)
Supplement: F1 [file EMS128764-supplement-F1.docx]

**Supporting Information to “All-systolic first-pass myocardial rest perfusion at a long saturation time using simultaneous multi-slice imaging and compressed sensing acceleration”**

Giulio Ferrazzi^1,2,*^, Sarah McElroy^1,*^, Radhouene Neji^1,3^, Karl P. Kunze^1,3^, Muhummad Sohaib Nazir^1^, [Peter Speier](https://jcmr-online.biomedcentral.com/articles/10.1186/s12968-018-0502-7#auth-3)^4^, [Daniel Stäb](https://jcmr-online.biomedcentral.com/articles/10.1186/s12968-018-0502-7#auth-5)^5^, Christoph Forman^4^, Reza Razavi^1^, Amedeo Chiribiri^1^, Sébastien Roujol^1^

^1^School of Biomedical Engineering and Imaging Sciences, Faculty of Life Sciences and Medicine, King’s College London, London, United Kingdom

^2^IRCCS San Camillo Hospital, Venice, Italy

^3^MR Research Collaborations, Siemens Healthcare Limited, Frimley, United Kingdom

^4^Cardiovascular MR predevelopment, Siemens Healthcare GmbH, Erlangen, Germany

^5^MR Research Collaborations, Siemens Healthcare Limited, Melbourne, Australia

* denotes equally contributing authors

**Dark rim simulation**

A forward simulation was set up to investigate the effect that a higher BM ratio has on dark rim artefacts through the Fourier truncation of k-space data. A synthetic phantom in short axis orientation representing the heart was generated in Matlab. To simulate realistic resolution levels, the in vivo data was reviewed, and it was decided that a lattice of 42x42 pixels was sufficiently large to contain the heart. The phantom data consists of two concentric circles representing blood and myocardial tissues. The blood signal was set to 100 (arbitrary unit) and it was assigned within the inner circle. The myocardial signals were instead referenced to the blood signal value using BM ratios of 5, 3 and 1.5 (see achieved range of BM ratios at peak blood signal enhancement in Figure 3C in the main manuscript). The ratio between inner vs. outer circle radiuses was set to 0.75.

To simulate dark rim artefacts, the phantom with a BM ratio of 5 was taken as the reference image. This was Fourier transformed to simulate k-space. Then, the high frequency content was symmetrically removed around the k-space centre. The amount of frequencies that were set to zero (50%) was decided upon verifying the presence of dark rim in image space without substantial image blurring. The remaining phantom data underwent an equivalent filtering procedure.

Our results are depicted below (first row) as Supporting Information Figure S1. The second row of the Figure shows horizontal signal profiles through the centre of the images.

Ringing effects can be observed at the endocardial border in all images (although ringing tends to be less pronounced for lower BM ratios). The perception of dark rim (yellow arrows), decreases as a function of the BM ratio since the myocardium signal is higher. Measurements performed on the signal profiles in the second row of the Figure, revealed a myocardial to dark rim contrast of 47%, 23% and 10% (BM ratios 5, 3 and 1.5).

**
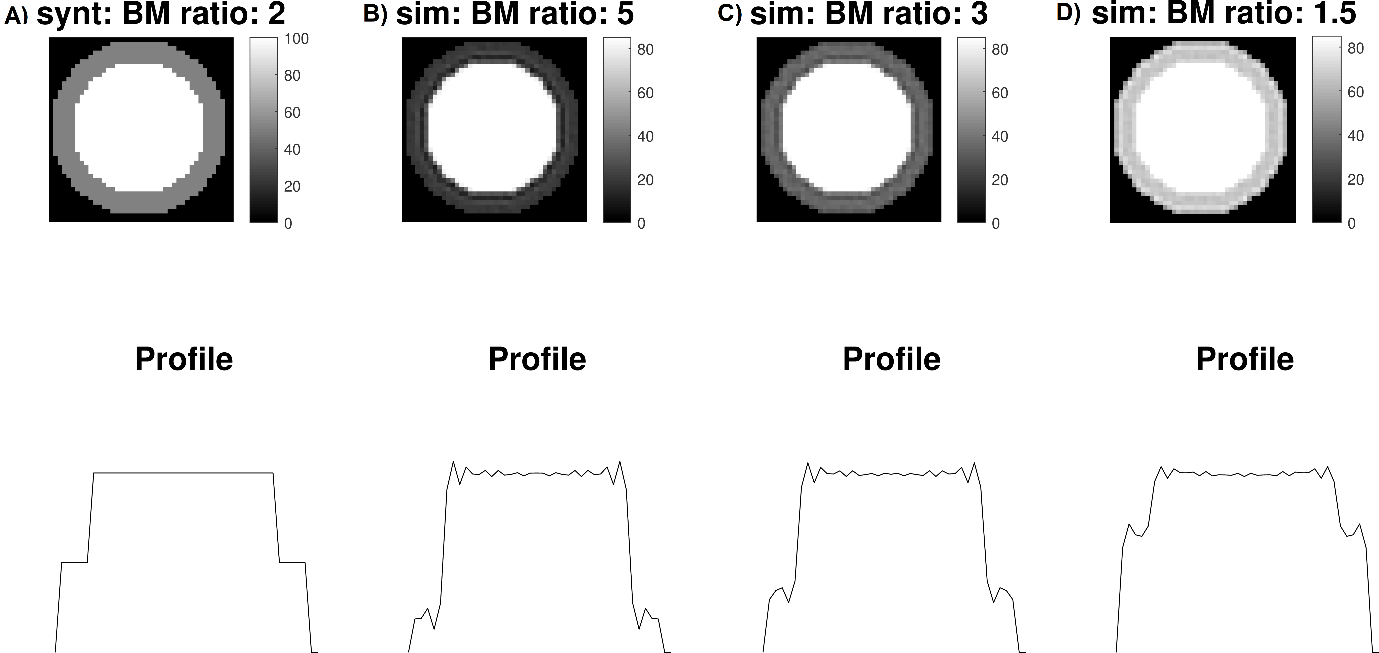
**

Supporting Information Figure S1: Dark rim simulation. A) Ground truth dataset resembling the heart in short axis orientation. B), C) and D) simulated phantom data with BM ratios of 5, 3 and 1.5. Bottom row, signal profiles through the center of the phantom.

**Scar patient examples**

Supporting Information Figure S2 reports the perfusion (A) and the Late Gadolinium Enhancement (LGE) positive (B) dataset acquired with SMS3 CS in a patient with chronic myocardial scar (0.05mmol/kg bolus injection). This can be observed in the anterior wall in the mid-ventricular slice and appears more conspicuous in LTS images than STS images (yellow arrow). LTS led to higher myocardium to scar contrast in the selected time frame (yellow box, magnified in Supporting Information Figure S2) with relative increase of 130%. Finally, note that there appears to be a lower intensity region on the lateral wall of the first few frames of the LTS data. Since there was not corresponding LGE in that region, this signal drop out is likely to be an artefact.


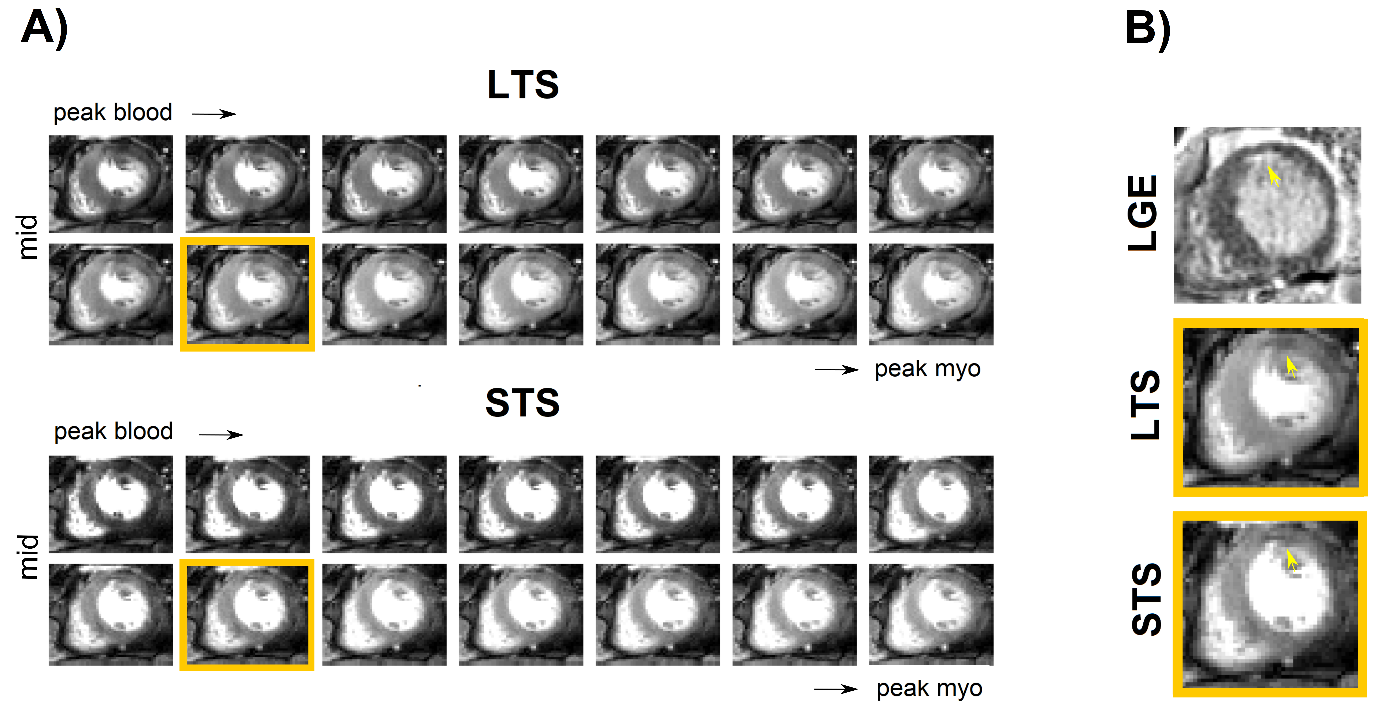


Supporting Information Figure S2: A) LTS (top) and STS (bottom) mid slice from the chronic scar patient acquired using SMS3 CS displayed from peak blood (top left image) to peak myo (bottom right). The yellow box highlights the frame magnified in B). B) LGE (top), LTS (center) and STS (bottom) scans highlighting the position of the scar (yellow arrow).

Supporting Information Figure S3 reports pilot data acquired on a scar positive patient (0.075mmol/kg bolus injection). This data used SMS3+TGRAPPA (1) acceleration but otherwise similar parameters to the SMS CS acquisition (LTS=300ms, STS=130ms, FOV=360x360mm^2^, slice thickness=10mm, resolution=2.3x2.3mm^2^, TR=2.56ms, TE=1.09ms, flip angle α=45^o^, readout bandwidth=1008Hz/Px, effective in-plane acceleration=2.3, readout duration=179ms). The resulting images are reported below. A Late Gadolinium Enhancement (LGE) image is also provided to highlight the position of the scar (yellow arrow). In each case, the data is displayed at peak myocardial signal enhancement and scaled according to the average signal within healthy myocardium. In this case, the scar lesion is more visible using a long saturation time than a short saturation time. When calculating myocardium to scar contrast, we observed an average increase of 73% when comparing LTS vs. STS images.


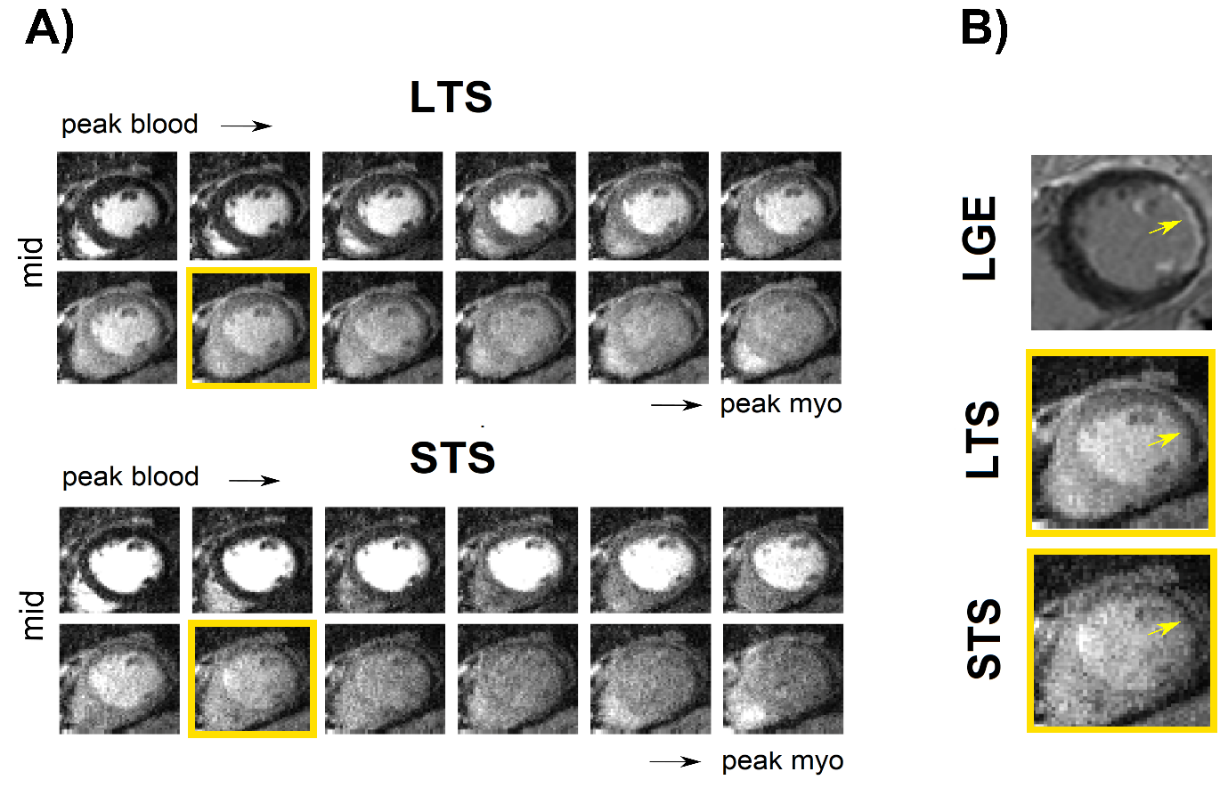


Supporting Information Figure S3: A) LTS (top) and STS (bottom) mid slice from the chronic scar patient acquired using SMS3 TGRAPPA acceleration displayed from peak blood (top left image) to peak myo (bottom right). The orange box highlights the frame magnified in B). B) LGE (top), LTS (center) and STS (bottom) scans highlighting the position of the scar (yellow arrow).

**REFERENCES**

1. Ferrazzi G, McElroy S, Radhouene. N et al. First-pass multi-contrast and multi-phase myocardial perfusion using simultaneous multi-slice imaging. In Proceedings of the 23th Annual Meeting of SCMR, Orlando, Florida 2020.
