# Supplementary material for: All-systolic first-pass myocardial rest perfusion at a long saturation time using simultaneous multi-slice imaging and compressed sensing acceleration
Source: Magn Reson Med. Author manuscript; Available in PMC 2022 Feb 1. (PMC7611406; doi:10.1002/mrm.28712)
Supplement: Supplementary Videos [file EMS128764-supplement-Supplementary_Videos.pdf]

FIGURE S3 A) LTS (top) and STS (bottom) mid slice from the chronic scar patient acquired using SMS3 TGRAPPA acceleration displayed from peak blood (top left image) to peak myo (bottom right). The orange box highlights the frame magnified in B). B) LGE (top), LTS (center) and STS (bottom) scans highlighting the position of the scar (yellow arrow)

VIDEO S1 Perfusion time series for the data presented in Figure 4. Vertical direction depicts LTS, STS and TGRAPPA datasets, whereas the horizontal direction shows base, mid and apical slices

VIDEO S2 Same as Supporting Information Video S1 for the data represented in Figure 6
